# Pancreaticoduodenectomy combined gastroduodenal collateral reconstruction and preservation due to median arcuate ligament syndrome: technical notes with two surgical cases report (with video)

**DOI:** 10.1186/s12957-023-03096-5

**Published:** 2023-07-17

**Authors:** Thanh Khiem Nguyen, Ham Hoi Nguyen, Tuan Hiep Luong, Pisey Chantha, Gia Khanh Ngo, Van Duy Le, Kim Khue Dang, Duc Huy Tran, Cuong Thinh Nguyen

**Affiliations:** 1 Department of Gastrointestinal and Hepato-Pancreato-Biliary Surgery, Bach Mai Hospital, Hanoi, Vietnam; 2 Department of Surgery, Hanoi Medical University, Hanoi, Vietnam; 3 Department of Thoracic & Vascular Surgery, Bach Mai Hospital, Hanoi, Vietnam; 4 VinUniversity, Ha Noi, Vietnam; 5 Center of Diagnostic Imaging and Intervention, Bach Mai Hospital, Hanoi, Vietnam; 6 108 Institute of Clinical Medical and Pharmaceutical Sciences, Hanoi, Vietnam

**Keywords:** Pancreaticoduodenectomy, Median arcuate ligament syndrome, Celiac axis stenosis, Gastroduodenal collateral preservation and reconstruction, Case report

## Abstract

**Introduction:**

Pancreaticoduodenectomy in patients with CA stenosis due to median arcuate ligament often required carefully collateral pathways management to avoid hepatic ischemic complications.

**Cases presentation:**

Case 1: A 63-year-old man was referred to our department because of jaundice with distal common bile duct tumor. Pancreaticoduodenectomy with left posterior SMA first approach and circumferential lymphadenectomy was performed. Case 2: A 48-year-old man was referred to our department because of right-upper-quadrant abdominal pain with Vater tumor. Laparoscopic pancreaticoduodenectomy with left posterior SMA first approach and circumferential lymphadenectomy was performed. Postoperatively, in all two cases, three-dimensional reconstruction images showed developed collateral pathways around the pancreatic head, and the CA was stenosis in 75% and 70% due to MAL, respectively. Intraoperatively, in all two cases, we confirmed poor blood flow in the common hepatic artery (CHA) by palpation and observation. So that in the first case, we have decided to proceed a no-touch technique of GDA segmental resection en bloc with the tumor and reconstructed with an end-to-end GDA anastomosis; in the second cases, we have decided to proceed gastroduodenal collateral preservation. When preserving these collateral pathways, we confirmed that the PHA flow remained pulsatile as an indicator that the blood flow was adequate.

**Conclusion:**

Celiac axis stenosis was a rare but difficult-to-managed condition associated with pancreaticoduodenectomy. Collateral pathways management depends on variety of collateral pathways.

**Supplementary Information:**

The online version contains supplementary material available at 10.1186/s12957-023-03096-5.

## Introduction

Hepatic ischemic complication is one of main causes that lead to lethal outcome after pancreaticoduodenectomy [1]. And most of cases with hepatic ischemic complications, which included acute liver failure, hepatic abscesses, necrosis of extrahepatic bile ducts, and anastomotic dehiscence, were caused by a condition of celiac axis (CA) stenosis [2]. In general population, CA stenosis occurs in about 10–25%, and most of them were asymptomatic [3]. Due to the stenosis, the common hepatic artery (CHA) occasionally narrow and so that, there been always have exists of arterial pancreatoduodenal abnormalities (arcade, dilatation, channels and/or aneurysms), mainly of them were collateral arteries connecting the gastroduodenal artery (GDA) with the superior mesenteric artery (SMA) or to a right hepatic artery (RHA) coming from the SMA [2]. Therefore, one of the most important points during pancreaticoduodenectomy with CA stenosis was determining guaranteed significant hepatic arterial flow.

Median arcuate ligament syndrome (MALS) is the most common cause of celiac axis (CA) stenosis [1]. Due to intraoperative MAL division was failure recanalization of the arterial flow in 30–40% of cases [1, 4, 5], arterial revascularization must be required in these cases. Herein, we report a case of a 63-year-old male patient with diagnosis of tumor of distal common bide duct with MAL syndrome and was treated with pancreaticoduodenectomy combined gastroduodenal collateral reconstruction. All our work has been reported in line with the CARE criteria and guidelines [6].

## Case presentation

### Case 1

**Fig. 1:**
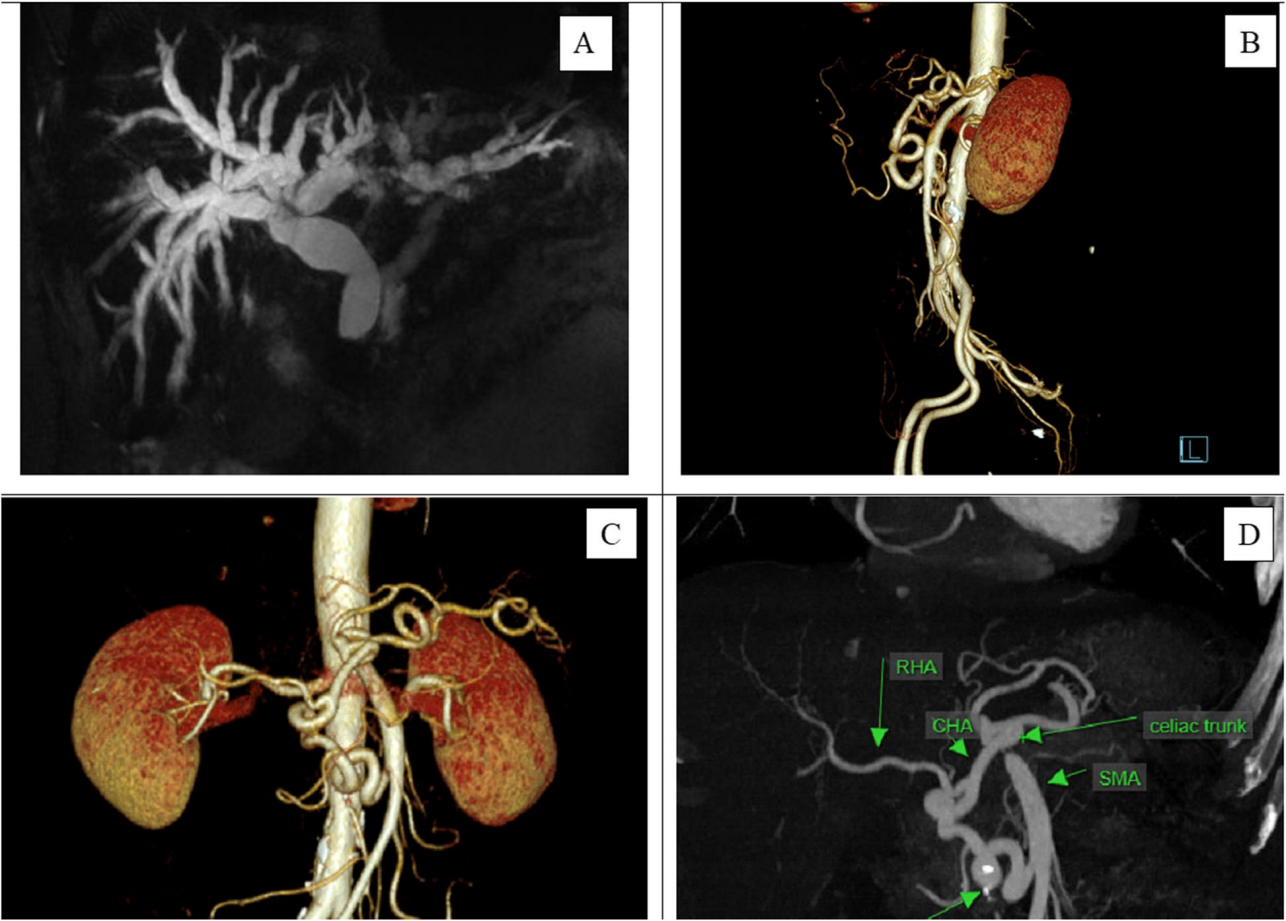
Gadoxetic acid-enhanced magnetic resonance imaging revealed a strict distal common bile duct with enhancement in the early phase (**A**). Three-dimensional reconstruction images showed developed collateral pathways around the pancreatic head (**B, C**). In the sagittal view, the CA was compressed severely by the MAL, which developed caudally (**D**) (CT, celiac trunk; CHA, common hepatic artery; GDA, gastroduodenal artery; IPDA, inferior pancreaticoduodenal artery; SMA, superior mesenteric artery; RHA, right hepatic artery)

A 63-year-old man was referred to our department with a distal common bile duct tumor. He had a history of 3-year diabetes with no surgical history. There was no family history of pancreatic cancer or genetic disorders. The patient’s appetite was normal, and he had no weight loss or abdominal pain, but jaundice increased in 2 weeks. Stool was normal with no history of constipation or diarrhea. Laboratory results included an elevated carbohydrate antigen 19–9 (CA19-9) level of 743.6 U/ml, but the carcinoembryonic antigen (CEA) level was normal. Other results, including diabetes indices such as HbA1c levels, and bilirubin levels, were elevated with a level of 308.1 μmol/l of total bilirubin and 173.7 μmol/l of directed bilirubin. Liver enzymes were also elevated, with alanine transaminase (ALT) and aspartate transaminase (AST) levels were 270 U/l and 111 U/l, respectively. In imaging examinations, gadoxetic acid-enhanced magnetic resonance imaging (MRI) revealed a strict distal common bile duct with enhancement in the early phase (Fig. 1A). The tumor had not invaded the major vessels, including the common hepatic artery (CHA), celiac axis (CA), and the superior mesenteric artery and vein (SMA and SMV), but metastatic lymph nodes (LNs) were apparent (LNs group numbers 8, 12, 7, and 14 according to Japanese Classification [7]). Three-dimensional reconstruction images showed developed collateral pathways around the pancreatic head (Fig. 1B, C). In the sagittal view, the CA was compressed by the MAL, which developed caudally (Fig. 1D). Endoscopic retrograde cholangiopancreatography (ERCP) revealed stenosis in the distal common bile duct and biopsy from the stenotic site-detected adenocarcinoma. He was diagnosed with cT2N2M0 (eighth edition of the UICC-TNM classification) distal common bile duct cancer co-morbid with CAS caused by MAL [8].

**Fig. 2:**
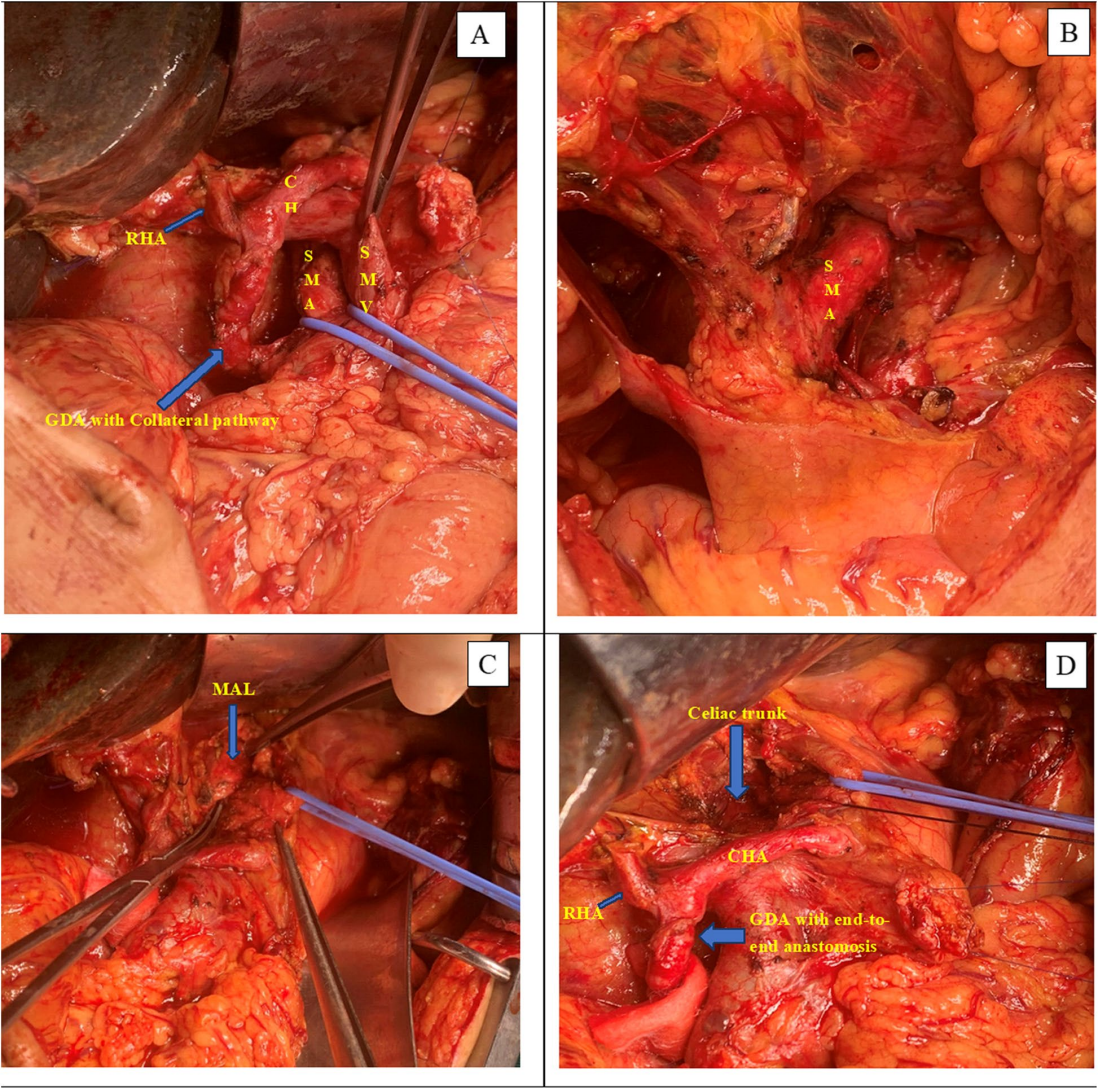
Pancreaticoduodenectomy with left-posterior SMA first approach and circumferential lymphadenectomy was performed (**A** and **B**). Median arcuate ligament before (**C**) and after (**D**) division (CT, celiac trunk; CHA, common hepatic artery; GDA, gastroduodenal artery; IPDA, inferior pancreaticoduodenal artery; SMA, superior mesenteric artery; RHA, right hepatic artery; MAL, median arcuate ligament)

A pancreaticoduodenectomy was planned because of the diagnosis of distal common bile duct tumor. After completing preoperative treatment, pancreaticoduodenectomy with left-posterior SMA first approach and circumferential lymphadenectomy was performed (Fig. 2). Since the CA flow was insufficient before the surgery, the surgical procedure was routinely performed, without any special preoperative arterial interventional techniques. After clamping the GDA and MAL division, we confirmed poor blood flow in the proper hepatic artery (PHA) by palpation, so that we have decided to resect a GDA segment that infiltrated to the tumor and proceeded end-to-end GDA anastomosis. With gastroduodenal collateral arcade was long enough, we have proceeded a no-touch technique with GDA segmental resection en bloc with the tumor. When preserving these collateral pathways, we confirmed that the PHA flow remained pulsatile as an indicator that the blood flow was adequate.

Postoperatively, there was a temporary elevation of the transaminase level, but that improved quickly, and there were no complications related to liver ischemia. Delayed gastric emptying (DGE) due to decreased gastric blood flow did not occur, and the patient was able to resume eating 4 days after the surgery [9]. The patient has not developed postoperative pancreatic fistula (POPF) based on the International Study Group of Pancreatic Surgery (ISGPS) grading [10]. The patient was discharged on postoperative day 12 with no major complications. The pathological diagnosis was pancreatic adenocarcinoma, pT3N1M0, and the cancer was stage IIB based on the eighth edition of the UICC-TNM classification with infiltrated tumor cell to GDA serosa [8]. No viable cancer cells were observed in the resected margin; thus, R0 resection was achieved. Due to postoperative staging, he had been referred to the oncological department for further assessment of adjuvant chemotherapy and follow-up.

### Case 2

A 48-year-old man was referred to our department with a Vater tumor. He had a history of 3-year diabetes with no surgical history. There was no family history of periampullary cancer or genetic disorders. The patient’s appetite was normal, and he had no weight loss or jaundice, but right-upper-quadrant abdominal pain increased in 2 weeks. Stool was normal with no history of constipation or diarrhea. Laboratory results included an elevated carbohydrate antigen 19–9 (CA19-9) level of 500.6 U/ml, but the carcinoembryonic antigen (CEA) level was normal. Other results, including bilirubin levels, were elevated with a level of 80.1 μmol/l of total bilirubin and 53.6 μmol/l of directed bilirubin. Liver enzymes were also elevated, with alanine transaminase (ALT) and aspartate transaminase (AST) levels were 172 U/l and 61 U/l, respectively. In imaging examinations, gadoxetic acid-enhanced magnetic resonance imaging (MRI) revealed a Vater tumor of diameter 15 × 9 mm with enhancement in the early phase (Video 1). The tumor had not invaded the major vessels, including the common hepatic artery (CHA), celiac axis (CA), and the superior mesenteric artery and vein (SMA and SMV), but metastatic lymph nodes (LNs) were apparent (LNs group numbers 8, 12, 7, and 14 according to Japanese classification [7]). Three-dimensional reconstruction images showed developed collateral pathways around the pancreatic head (Video 1). In the sagittal view, the CA was compressed about 70% by the MAL, which developed caudally (Video 1). He was diagnosed with cT2N0M0 (eighth edition of the UICC-TNM classification) of Vater cancer co-morbid with CAS caused by MAL [8].

A laparoscopic pancreaticoduodenectomy was planned because of the diagnosis of Vater tumor. After completing preoperative treatment, laparoscopic pancreaticoduodenectomy with left posterior SMA first approach and circumferential lymphadenectomy was performed following our technique (Video 1) [11]. Since the CA flow was insufficient before the surgery, the surgical procedure was routinely performed, without any special preoperative arterial interventional techniques. After clamping the GDA and MAL division, we confirmed poor blood flow in the proper hepatic artery (PHA) by palpation, so that we have decided to preserve the gastroduodenal collateral arcade (GCA), and then we have proceeded a no-touch technique with GCA preservation. When preserving these collateral pathways, we confirmed that the PHA flow remained pulsatile as an indicator that the blood flow was adequate.

**Fig. 3:**
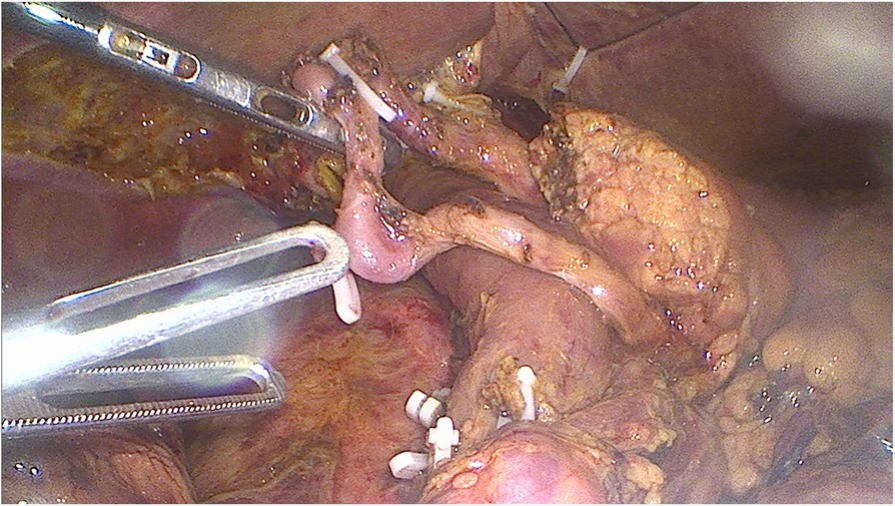
Laparoscopic pancreaticoduodenectomy with left-posterior SMA first approach, circumferential lymphadenectomy, and gastroduodenal collateral arcade (GCA) preservation was performed

In all two cases, we attempted to dissect the main collateral circulation from the pancreatic head parenchyma, mainly using the LigaSure™ for good bleeding control. We only focus on preserving the main collateral circulation to be sure to maintain the blood supply to the liver. The rest, during the dissection of the collateral circulation, we will ligate all the remaining arterial branches. Alternatively, it may be possible with LigaSure™ or a Hem-o-lok™ clamp for large arterial branches greater than 5 mm (Fig. 3).

Postoperatively, there were no complications related to liver ischemia. No delayed gastric emptying (DGE) due to decreased gastric blood flow has occurred, and the patient was able to resume eating 4 days after the surgery. The pathological diagnosis was pancreatic adenocarcinoma, pT2N0M0 (total 35 negative lymph nodes), and the cancer was stage IIA based on the eighth edition of the UICC-TNM classification [8]. No viable cancer cells were observed in the resected margin; thus, R0 resection was achieved. He has recharged after 20 days of post-operation.

## Discussion and conclusion

Celiac axis (CA) stenosis was reported to be found in about 5–10% of patients who underwent PD [12, 13]. In diagnosis, a preoperative multidetector computed tomography (CT) with routine three-dimensional arterial reconstruction was gold standard [14]. According to Smith et al., stenosis smaller than 60% of CA diameter has not caused an increased risk of postoperative or perioperative complications in patients undergoing PD [15], and with patients of arterial stenosis of < 50%, decompression procedures were not required before or during PD [13]. However, severe CA stenosis must require revascularization surgery and/or endovascular stenting [13].

Along with atherosclerosis, compression by the MAL is accounted for about 90% cause of severe CAS [1]. The median arcuate ligament runs transversely to the origin of the CA proximally, to the L1 vertebral body anteriorly, and to the abdominal aorta ventrally. While MAL division is simple, in some cases, it is not enough because the inability of CA recanalization and hepatic blood flow is not secured. In totally 108 cases of patients who underwent pancreaticoduodenectomy with MAL syndrome that could be found in English literature, there were 21.2% cases that hepatic arterial flow could not be restored after MAL division. And according to Bong et al., the MAL division was not required in all cases but in cases that the collateral pancreatoduodenal arteries (PDAs) could not be saved due to technical and oncological demands [16].

The CA stenosis usually led to collateral pancreatoduodenal arteries to supply hepatic blood flow. According to a systematic review by Giovanardi, Lai, Garofalo, Arroyo Murillo, Choppin de Janvry, Hassan, Larghi Laureiro, Consolo, Melandro, and Berloco, in totally 87 cases of patients who underwent pancreaticoduodenectomy with CAS syndrome that they could found in English literature, PDAs abnormalities were reported in 73 cases (83.9%). And among them, 67 cases had a real anterior and/or posterior arcade between GDA and SMA, but enlarged GDA was observed in only four cases [3]. In our case, CA stenosis was diagnosis preoperatively, and a collateralization of GDA dilation with SMA was found. There were other arterial abnormalities like dorsal pancreatic artery (DPA)-RHA channel, DPA-splenic artery (SA) channel, middle colic artery (MCA)-PD arcade channel, SMA-SA channel via the MCA, and/or an accessory or replaced RHA. In our case, beside narrow CHA, there were an accessory left hepatic artery (LHA) came from left gastric artery (LGA).

Due to the failure of MAL division can only be determined intraoperatively, and in these situations, surgeons must make a choice, with complex revascularization or abandonment of the surgery. However, with the development of diagnostic imaging therapies preoperatively like CT with routine three-dimensional arterial reconstruction or preoperative endovascular intervention, the colleterial arterial pathways can be determined very clearly. So that, it is not impossible for surgeons to determine the ability of radical resection with collateral pathway management without impairing the curativeness of cancer. In some research, authors have chosen a preoperative management of CAS, and one of the most popular choice was CA preoperative endovascular stenting [17, 18], but this technique had a risk of failure due to persistent external CA compression as well as had an inferior long-term longevity compared with arterial reconstruction [4]. In addition, it has taken time to observe the affection of CA recanalization after stenting, and double-antiplatelet therapy (DAPT) was subsequently administered to avoid stent obstruction according to antithrombotic therapy guidelines for patients after percutaneous coronary intervention [19]. But the optimal time between stent insertion and radical surgery has not yet been investigated. In these cases, some authors have chosen neoadjuvant chemotherapy (NAC) to administer the development of tumor. However, in diseases where NAC is not a common treatment, such as biliary tract cancer, wasting time could lead to worsen the primary disease.

In our two cases, a collateralization of GDA dilation with SMA was determined preoperatively, and we have not chosen CA preoperative endovascular stenting with these above reasons. Intraoperatively, after GDA clamping and MAL division, the hepatic arterial flow through CHA has not recovered, so we have decided to resect a GDA segment that infiltrated to the tumor and proceeded end-to-end GDA anastomosis. Postoperatively, patient was administered by continuous intravenous (IV) infusion unfractionated-heparin therapy following the American Heart Association (AHA) guideline [20]. One of an important technical point is that, in the first case, due to the occasional infiltration of tumor to the GDA serosa as well as to avoid bleeding from enlarged dorsal pancreatic branches of PDAs, we recommend proceeding a no-touch technique of GDA segmental resection en bloc with the tumor, in order to avoid bleeding from enlarged dorsal pancreatic branches from PDAs and reconstructed with an end-to-end GDA anastomosis if the gastroduodenal collateral arcade was long enough, like in our case. In the second case, intraoperative observation has shown no occasional infiltration of tumor to the GCA, so we have decided preserving the GCA laparoscopically to avoid hepatic ischemia.

## Conclusion

Celiac axis stenosis was a rare but difficult-to-managed condition that can occur in patients who underwent pancreaticoduodenectomy. If median arcuate ligament division does not restore hepatic arterial flow, gastroduodenal collateral arcade reconstruction and preservation are needed. Collateral pathways management depends on variety of collateral pathways; in some cases, gastroduodenal artery resection and reconstruction with end-to-end anastomosis with a no-touch technique were recommended to minimize risks of complicated revascularization.

## Supplementary Information

**Additional file 1: Video 1.** Laparoscopic pancreaticoduodenectomy with left-posterior SMA first approach and circumferential lymphadenectomy was performed (CT: Celiac trunk, CHA: Common hepatic artery, GDA: Gastroduodenal artery, IPDA: Inferior pancreaticoduodenal artery, SMA: Superior mesenteric artery, RHA: Right hepatic artery, MAL: Median arcuate ligament).

## Data Availability

All data generated or analyzed during this study are included in this published article.

## Abbreviations

CA: Celiac artery
CEA: Carcinoembryonic antigen
CHA: Common hepatic artery
CT: Computed tomography
EUS: Endoscopic ultrasound
FJA: First jejunal artery
FJV: First jejunal vein
GDA: Gastroduodenal artery
IPDA: Inferior pancreaticoduodenal artery
IPDV: Inferior pancreaticoduodenal vein
LN: Lymph nodes
LRV: Left renal vein
NCCN: National Comprehensive Cancer Network
MAL: Median arcuate ligament
MRI: Magnetic resonance imaging
PDAC: Pancreatic ductal adenocarcinoma
POPF: Postoperative pancreatic fistula
PPDA: Posterior pancreaticoduodenal artery
PD: Pancreaticoduodenectomy
SMA: Superior mesenteric artery
SMV: Superior mesenteric vein
RGA: Right gastric artery
